# Kindler Syndrome in a 24-Year-Old Male: A Clinical Diagnosis in the Absence of Genetic Testing: A Rare Case Report

**DOI:** 10.1155/crdm/9343494

**Published:** 2025-10-28

**Authors:** Dyala Sayed Ahmad, Rim Nasser, Alaa Mahmoud, Moatasem Hussein Al-Janabi, Zuheir Al-Shehabi, Fouz Hassan

**Affiliations:** ^1^Department of Dermatology and Sexually Transmitted Disease, Tishreen University Hospital, Latakia, Syria; ^2^Department of Pathology, Cancer Research Center, Tishreen University Hospital, Latakia, Syria; ^3^Department of Pathology, Director of Cancer Research Center, Tishreen University Hospital, Latakia, Syria

**Keywords:** blisters, epidermolysis bullosa, genodermatosis, kindler syndrome

## Abstract

Kindler syndrome is an uncommon autosomal recessive genodermatosis, with roughly 400 documented cases worldwide as of March 2024. We describe a 24-year-old male, born to consanguineous but otherwise healthy parents, who presented with photosensitivity, gingival fragility, thinning of the skin, restricted finger mobility, and tooth loss. Although molecular testing represents the standard diagnostic tool, our diagnosis was established on the basis of distinctive clinical and histopathological features, which remain crucial in settings where genetic confirmation is unavailable.

## 1. Introduction

Kindler syndrome (KS) is a rare autosomal recessive disorder of the skin first reported by Theresa Kindler in 1954 [1]. It represents a subtype of inherited epidermolysis bullosa (EB) caused by mutations in the Fermitin family homologue 1 (FERMT1) gene, which encodes kindlin-1. This protein is expressed primarily in the skin, periodontal tissue, and intestinal epithelium [2].

Clinically, KS manifests with acral blistering and hemorrhagic vesicles during infancy, progressing to skin atrophy resembling cigarette-paper skin and poikiloderma [3]. Additional cutaneous findings include diffuse palmoplantar hyperkeratosis and pseudosyndactyly [4]. Mucosal involvement is common and may lead to periodontal disease, premature tooth loss, hemorrhagic mucositis, labial leukokeratosis, ectropion, and urethral stenosis [1]. Long-term complications include strictures, periodontitis, and an increased risk of squamous cell carcinoma (SCC) [5].

Diagnosis is typically based on clinical features and confirmed by genetic testing [2]. However, in resource-limited settings, clinical criteria remain the mainstay of diagnosis. We present the clinical course and histopathological findings of a 24-year-old male with KS, diagnosed in the absence of molecular confirmation.

## 2. Case Presentation

A 24-year-old man, the product of a consanguineous union, presented with photosensitivity, skin fragility, and restricted finger mobility. He also reported multiple missing teeth and severe gingivitis. Additional complaints included dysphagia and reduced mouth opening.

According to his mother, blistering had begun in infancy and recurred frequently throughout childhood, primarily in acral regions after minor trauma or sun exposure. Lesions typically healed with atrophic scarring, milia-like papules, and mottled hypo- and hyperpigmentation. Each episode was treated conservatively with topical antiseptics, emollients, or wound care. Despite partial resolution, new lesions developed repeatedly. His 19-year-old sister and a cousin had similar symptoms.

**Figure 1:**
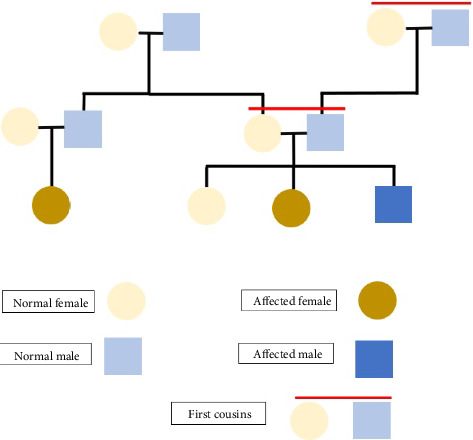
Pedigree chart showing a three-generation family with consanguinity and affected members, illustrating the autosomal recessive inheritance pattern.

A three-generation pedigree revealed consanguinity: the patient's parents are first cousins, and the paternal grandparents are also first cousins. The patient has two sisters, one affected by KS, and a maternal cousin is similarly affected. Parents are asymptomatic obligate heterozygous carriers of an autosomal recessive mutation. The pedigree chart illustrating consanguinity and affected family members is shown in (Figure 1).

**Figure 2:**
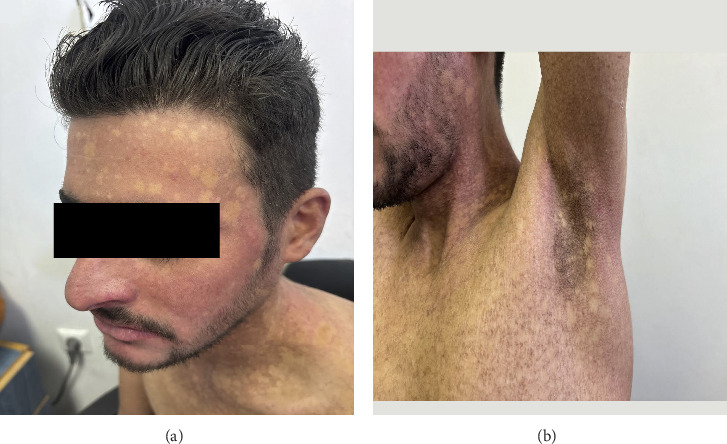
Clinical images. (a) Hypopigmented macules on the face and neck. (b) Areas of hyperpigmentation and hypopigmentation in the axilla.

**Figure 3:**
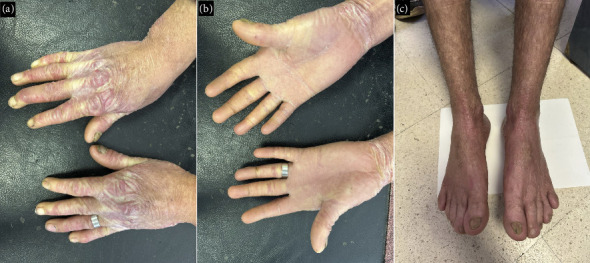
Clinical images. (a) Atrophy and wrinkling of the dorsal hand skin (cigarette-paper scarring) with thickened cuticles and limited finger extension. (b) Waxy keratoderma with dermatoglyphic patterns on the palms. (c) Nail dystrophy and skin atrophy of the feet.

Physical examination revealed generalized poikiloderma with widespread hypo- and hyperpigmented patches over the face, neck, axillae, and extremities (Figure 2). The skin was thin, wrinkled, and atrophic, particularly on the hands and feet, resulting in loss of fingerprints and impaired finger movement. Additional findings included nail dystrophy, erythematous scaly plaques on flexor surfaces, digital banding, and localized hyperkeratosis (Figure 3).

**Figure 4:**
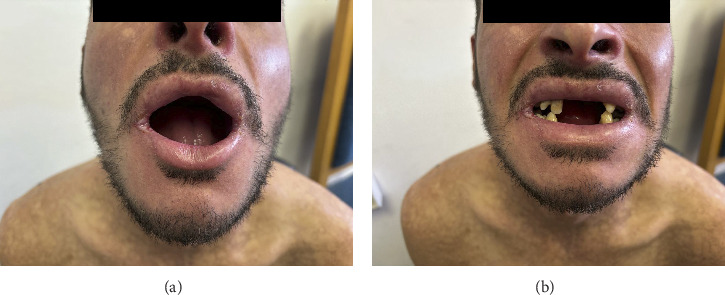
Clinical images. (a) Limited oral opening and angular cheilitis. (b) Loss of teeth.

Oral examination demonstrated chronic periodontitis, gingivitis, recurrent mucosal bleeding, multiple missing teeth, and restricted mouth opening. This limitation was attributed to a combination of mucosal fragility, perioral scarring with contractures, reduced elasticity of tissues, and chronic inflammation affecting temporomandibular joint function. Hyperkeratotic plaques were also noted on the oral and genital mucosa (Figure 4).

Ocular findings were unremarkable, and routine laboratory investigations were within normal limits. Genetic testing for KS was unavailable in the local setting.

**Figure 5:**
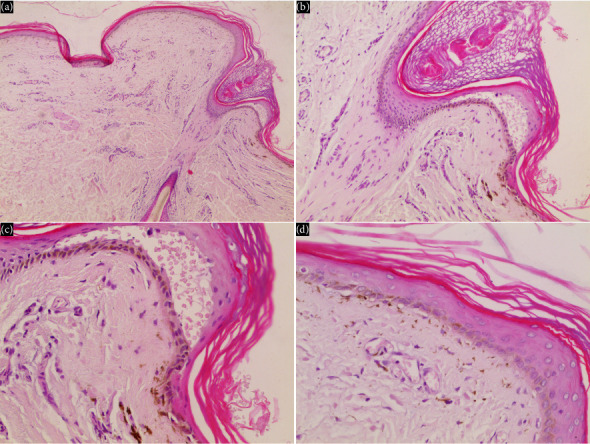
Histopathological features of the skin biopsy (hematoxylin and eosin stain). (a) Low-power view (40x) showing epidermal atrophy and subepidermal cleft formation. (b) Low-power view (100x) highlighting focal vacuolar degeneration of the basal layer and overlying epidermal thinning. (c) High-power view (200x) revealing pigmentary incontinence with melanin deposition in the dermis. (d) High-power view (400x) demonstrating dilated superficial dermal blood vessels.

Histological findings: An incisional biopsy from the leg showed epidermal atrophy, dilated superficial dermal vessels, focal vacuolar degeneration at the basal layer, subepidermal cleft formation, and pigmentary incontinence (Figure 5). The biopsy was taken when the patient was 24 years old, thus representing a late stage of the disease. Accordingly, the histological picture reflected chronic changes. While these findings are supportive, they are not pathognomonic for KS and can be seen in other hereditary bullous poikiloderma syndromes.

Based on the combination of recurrent blistering in infancy, progressive poikiloderma, nail dystrophy, severe gingival involvement, mucosal fragility, and supportive histopathological changes, a diagnosis consistent with KS was established.

## 3. Discussion

KS remains a rare disorder, with fewer than 400 reported cases worldwide [1]. It is characterized by trauma-induced acral blistering, photosensitivity, and progressive poikiloderma, typically manifesting early in life and often stabilizing in adolescence. Our patient also demonstrated axillary freckles, a relatively uncommon feature, in addition to palmoplantar hyperkeratosis, nail dystrophy, and pseudosyndactyly.

Mucosal disease is a major contributor to morbidity, with strictures and leukokeratosis leading to dysphagia, poor oral health, and risk of malignancy. Patients are predisposed to SCC, especially of the lip and palate [1]. The condition is more frequently described in individuals of Arab, Iranian, Turkish, Indian, and European ancestry [4]. Differential diagnoses include Rothmund–Thomson syndrome, Bloom syndrome, and Cockayne syndrome [4].

Histopathological features of KS are variable and largely nonspecific. Epidermal atrophy, vacuolar degeneration, and subepidermal clefting can be seen in KS but are not exclusive and depend on the lesion sampled. Similar changes occur in other hereditary bullous poikiloderma syndromes. For this reason, diagnosis in our case was based not solely on histology, but on a comprehensive evaluation incorporating clinical criteria, family history of consanguinity with similarly affected relatives, and supportive biopsy findings. This integrative approach is in line with the diagnostic framework proposed by Fischer et al. [6].

In 2005, Fischer et al. [6] proposed clinical diagnostic criteria for KS based on major, minor, and associated findings (Table 1). The major criteria are acral blistering in infancy and childhood, progressive poikiloderma, abnormal photosensitivity, skin atrophy, and gingival fragility or swelling. Minor criteria include syndactyly and mucosal involvement such as urethral, anal, esophageal, and laryngeal stenosis. Associated findings are nail dystrophy, ectropion of the lower lid, palmoplantar keratoderma, pseudoainhum, leukokeratosis of the lips, SCC, anhidrosis/hypohidrosis, skeletal abnormalities, and poor dentition or periodontitis [4]. Diagnosis is considered “certain” with four major criteria, “probable” with three major and two minor criteria, and “likely” with two major and two minor or associated symptoms [1]. The primary method for diagnosis is genetic analysis of mutations in the FERMT1 gene. The clinical criteria are used when genetic testing is unavailable, as in our case.

This case met five major diagnostic criteria, two minor criteria, and four associated findings. The patient experienced moderate dysphagia to solids due to oral and esophageal lesions.

There is no definitive therapy for KS. Management focuses on wound care, photoprotection, prevention of secondary infections, and symptomatic relief [3]. Genetic counseling is essential. Our patient and his affected sister were counseled on disease inheritance, prognosis, and the importance of regular dermatological follow-up for early detection of neoplasia. Despite the generally poor long-term outlook [3], this patient remains in stable health and has a healthy child from a nonconsanguineous marriage.

Diagnostic Criteria for Kindler Syndrome-Table 1.

## 4. Conclusion

KS is a rare and heterogeneous disorder with significant clinical implications. In the absence of molecular confirmation, diagnosis can still be achieved through detailed clinical evaluation, family history, and supportive histological features. This case illustrates the importance of awareness and early recognition of KS, particularly in regions with limited access to genetic testing. Comprehensive management strategies focusing on prevention, symptomatic care, and long-term surveillance can help improve quality of life. Further studies and collaborative efforts are needed to enhance diagnostic accuracy and develop targeted therapeutic options.

## Figures and Tables

**Table 1:** Summarizes the diagnostic criteria met by the 24-year-old patient.

Criteria type	Specific finding	Present in patient
Major	Acral blistering in infancy/childhood	✓
	Progressive poikiloderma	✓
	Photosensitivity	✓
	Skin atrophy	✓
	Gingival fragility/swelling	✓

Minor	Syndactyly	✓
	Mucosal involvement (oral/esophageal/urethral)	✓

Associated	Nail dystrophy	✓
	Pseudoainhum	—
	Palmoplantar keratoderma	✓
	Leukokeratosis of the lips	✓

## Data Availability

The data that support the findings of this study are available from the corresponding author upon reasonable request.
